# Hospital Sunday

**Published:** 1896-06-13

**Authors:** Walter Wilkin

**Affiliations:** Lord Mayor. The Mansion House, London


### Editor's Letter-Box.

### HOSPITAL SUNDAY.

Sir,—The twenty-fourth anniversary of "Hospital Sunday" in London falls on Sunday next, and as president and treasurer of the Fund I invite your courteous assistance in giving publicity to the annual appeal which each successive Lord Mayor has felt it his duty to make since the movement was initiated at the Mansion House.

At no period in the history of the Fund were the claims upon it more urgent. Last year the voluntary hospitals and medical charities relieved over 1,463,000 patients at a cost of £718,264. The ordinary income of these institutions only amounted to £576,447, leaving a deficit of £141,817. The receipt of substantial legacies enabled some of the hospitals to balance the year's accounts, but the majority of the institutions remain in debt, anxiety, and difficulty. But the most serious point is that even these results were only obtained through a compulsory abandonment, for want of funds, of a large portion of the work of which the hospitals of London are capable. This is seen from the fact that while the number of beds available for the sick and suffering poor is 9,005, only 6,351 were able to be used last year, leaving no fewer than 2,654 empty, solely for lack of the resources necessary to maintain and restore to health the patients for whom they were intended.

This year the Hospital Sunday Fund has applications from 127 hospitals and 54 dispensaries to participate in the collections to be raised on Sunday, and it is estimated that at least £100,000 will be required to meet their pressing needs. The progress of the Fund has been continuous and gratifying. In 1873 the receipts were £27,700. Last year they reached a total of over £60,000, but of this £15,000 was the result of a special collection on the Stock Exchange, and a few munificent offerings which this year will be missing. In all, £828,697 has been raised on Hospital Sunday in the past twenty-three years, and the expenses of collection have averaged only 2·6 per cent. The increase in the contributing congregations has been equally striking, for these have risen from 1,072 in 1873 to 1,817 at the present time, and few congregations now omit to take part in the movement.

These figures establish that, while much has been done in the past, the need of providing for the requirements of the sick poor of London is increasingly and pressingly growing, and that the necessity for enhanced liberality on Hospital Sunday becomes correspondingly great. Any diminution of public sympathy in this movement can only result in a further reduction of hospital usefulness and an addition to the already large number of empty beds and disused wards.

To dwell in detail on the work done in the hospitals would be wearisome, but if the record of patients attended is insufficient to commend them to public support, I would allude to those other aspects in which they may be regarded—as the great training schools for medical men and skilled nurses, and their use in the discovery and the application of scientific methods for the prevention and treatment of prevalent disease inseparable from a huge city like London, where multitudes of people are forced to live in unhealthy, injurious, and insanitary surroundings.

"Hospital Sunday" has another beneficent feature. It is the one day in all the year in which people sink their religious differences and combine in a solemn act of devotion and charity in behalf of their sick, suffering, and dying fellow-creatures. To co-operate in so beneficent a work is indeed a privilege, and I venture to think that "Hospital Sunday" may, under the Divine blessing, not only have the direct result of largely benefiting the hospitals, but produce such feelings of unity and goodwill as can not fail to have a wide influence in our religious, social, and charitable work.—I have the honour to be, Sir, your obedient servant,

Walter Wilkin, Lord Mayor.

The Mansion House, London, June 11th, 1896.

P.S.—Contributions of those who may be prevented from sending their donations through a place of worship will be gratefully received by Mr. H. N. Custance, the secretary of the Fund, at the Mansion House.

